# Mast cells partially contribute to mucosal adjuvanticity of surfactin in mice

**DOI:** 10.1002/iid3.204

**Published:** 2017-11-03

**Authors:** Naoto Yoshino, Ryosuke Takeshita, Hanae Kawamura, Yutaka Sasaki, Masahiro Kagabu, Toru Sugiyama, Yasushi Muraki, Shigehiro Sato

**Affiliations:** ^1^ Division of Infectious Diseases and Immunology Department of Microbiology, School of Medicine, Iwate Medical University Iwate Japan; ^2^ Department of Obstetrics and Gynecology School of Medicine, Iwate Medical University Iwate Japan

**Keywords:** adjuvants, body temperature, histamine, lipopeptides, mast cells

## Abstract

**Introduction:**

Surfactin (SF) is a cyclic lipopeptide that has potent mucosal adjuvant properties. However, immunological mechanisms of SF adjuvant action have not yet been elucidated. As some cyclic lipopeptides, such as polymyxin, can stimulate histamine release from mast cells, we hypothesized that mast cell activation is critical for SF adjuvanticity.

**Methods/Results:**

We observed that following intranasal immunization with ovalbumin (OVA) plus SF, the titers of the OVA‐specific antibody (Ab) in the mucosal secretions and plasma of mast cell‐deficient mice were significantly lower than those in congenic normal mice, although OVA‐specific Ab did not entirely disappear from mast cell‐deficient mice. SF induced degranulation of mast cells and release of histamine in vitro. To investigate whether SF stimulated mast cells in vivo, we measured body temperature of mice immunized intranasally with OVA plus SF because histamine level affects body temperature. Following immunizations, body temperature of immunized congenic normal mice transiently decreased, whereas body temperature of mast cell‐deficient mice did not change. Plasma levels of OVA‐specific IgE Ab were not significantly different in mast cell‐deficient and congenic normal mice. These findings suggest that SF directly affected mast cells in an IgE Ab‐independent fashion. Furthermore, we analyzed the effects of SF on MC/9 mast cells cultured in vitro. MC/9 cells stimulated by SF released not only histamine but also leukotriene B_4_ and prostaglandin D_2_. Moreover, SF up‐regulated mRNA expression levels of *Tnf*, *Ccr5*, and *Il4* genes in mast cells. These cytokines may play a facilitating role in OVA‐specific immune responses in mice.

**Conclusion:**

Overall, our results showed that mast cell activation partially mediated SF adjuvanticity.

## Introduction

Mucosal tissues, such as nasal cavity, trachea, oral cavity, gastrointestinal tract, and urogenital apparatus, are points of pathogen entry and therefore, they represent the biophylaxis frontline. Mucosal vaccines can enhance immunocompetence for mucosal defense and prevent the invasion of pathogens 1, 2. However, the addition of mucosal adjuvant is usually required to induce a strong immune response against vaccine antigens (Ags) and, subsequently, against mucosal infection.

Several recent studies have shown that mast cells play an active role in the development of adaptive immunity. As mast cells affect various immune cells by releasing cytokines and chemical mediators 3, mast cell activators induce robust Ag‐specific immune responses when co‐administered with vaccine Ags 4, 5, 6. For example, compound 48/80, a mast cell activator, enhanced humoral immunity more powerfully in wild‐type mice than in mast cell‐deficient mice 6. This substance has been examined as an adjuvant of vaccines against botulinum neurotoxin, anthrax, and *Streptococcus pneumonia* 7, 8, 9. Therefore, mast cell activators are considered a novel class of vaccine adjuvants 6.

Polymyxin B (PMB) and colistin (CL) have been shown to activate mast cells 10, 11, 12. PMB and CL, isolated from *Paenibacillus polymyxa* 13, 14, are well‐known antibiotics commonly used in the clinical setting. It has been shown that these compounds exhibited mucosal adjuvanticity 12. In particular, potent adjuvanticity of PMB stemmed from its propensity to activate mast cells 12.

Recently, it has been demonstrated that compounds of the surfactin (SF) family 15, such as SF and WH1fungin, have potent mucosal adjuvanticity 16, 17, 18. SF and WH1fungin were isolated from *Bacillus subtilis* 19 and *B. amyloliquefaciens* 20, respectively. Intranasal immunization with SF and ovalbumin (OVA) enhanced OVA‐specific humoral immune responses in a dose‐dependent manner at both mucosal and systemic compartments 16. However, whether SF, as a mucosal adjuvant, enhances protective efficacy of Abs against pathogens has not yet been investigated. WH1fungin facilitated cytotoxic T lymphocyte responses to OVA and hepatitis B surface Ag after oral and intranasal immunizations 17, 18. However, in vivo immunological mechanisms that confer mucosal adjuvant properties on SF are still unknown.

SF is a much more potent mucosal adjuvant than PMB and CL, and, probably, most promising adjuvant among the cyclic lipopeptides 12, 16. Therefore, elucidating the immunological mechanism of SF adjuvanticity is important. We noted that SF, PMB, and CL share a common structural element consisting of a cyclic heptapeptide bound to a fatty acid chain 21, 22, 23. Because the structure of SF is similar to those of PMB and CL, it was expected that SF would activate mast cells and, as a result, enhance Ag‐specific immune responses. Therefore, to study the mechanism of SF adjuvanticity, we focused on mast cells and explored whether their activation accompanied SF adjuvanticity manifestations. We revealed that SF directly affected murine mast cells in vivo in an IgE Ab‐independent fashion. We also showed that SF activated murine MC/9 mast cells cultured in vitro and increased expression levels of several cytokines.

## Materials and Methods

### Animals

Mast cell‐deficient WBB6F1/kit‐*Kit* ^w^/*Kit* ^w‐v^ (*W/W^v^*) mice carrying mutations at the *W* locus (dominant white spotting) were used in this study 24, 25, 26. Female 5‐week‐old *W/W^v^* mice and their mast cell‐sufficient WBB6F1‐^+/+^ littermates (congenic normal) were purchased from Japan SLC, Inc. (Yokohama, Japan). The mice were acclimated to the experimental animal facility for more than a week before being used in experiments and maintained under pathogen‐free conditions. Mice were housed in groups of four in plastic cages (225 × 338 × 140 mm^3^) with a stainless‐steel grid lid and wood shavings scattered on the floor. The vivarium room had a temperature of 22 ± 1°C and was maintained at 12:12‐h light/dark cycle with lights off at 7 p.m. The mice were fed with pelleted MF (Oriental Yeast Co., Ltd., Tokyo, Japan) and had ad libitum access to deionized water.

### Ethics statement

All animal protocols adhered to the recommendations of the Guidelines for Proper Conduct of Animal Experiments established by the Science Council of Japan and were approved by the Committee on the Ethics of Animal Experiments (CEAE) at the Iwate Medical University. All animal experiments were performed in accordance to the guidelines set by Iwate Medical University CEAE (permit numbers 24–016 and 26–037).

### Cells

MC/9 mast cells from mouse fetal liver (American Type Culture Collection, Manassas, VA, USA, catalog number: CRL‐8306) 27, which are typical cells for mast cell‐activating agent screening 28, were used for in vitro experiments. The maintenance of MC/9 cells was described elsewhere 12.

### Ag and adjuvant

Ovalbumin (OVA; purity ≥ 98%, Grade VI; Sigma–Aldrich, St. Louis, MO, USA) was employed as the model antigenic protein. The adjuvant SF sodium salt was obtained from Wako Chemical Industries, Ltd. (Osaka, Japan). Endotoxin concentration was determined to be <0.1 endotoxin units (EU)/mg of SF, using a Chromogenic Limulus Amebocyte Lysate Endotoxin Assay Kit (GenScript, Piscataway, NJ, USA).

### Immunization of mice

To prepare the immunization solution, OVA and SF were dissolved in normal saline (<0.25 EU/mL; Otsuka Pharmaceutical Factory, Inc., Tokushima, Japan). Mice, lightly anesthetized with ketamine, were intranasally immunized with 5‐μL aliquots (2.5 μL/nostril) of normal saline containing 100 μg of OVA with or without 500 μg of SF. All groups of mice were immunized three times at weekly intervals, according to the schedule that was largely the same as that used in previous studies 12, 29, 30.

### Assessments of OVA‐specific antibodies

Fecal extracts, nasal washes, saliva, and plasma were collected 1 week after the last immunization by the methods described elsewhere 31, 32. Titers of OVA‐specific antibodies (Abs) in mucosal secretions and plasma were determined by the endpoint enzyme‐linked immunosorbent assay (ELISA) 30. For IgG subclass analysis, OVA‐specific IgG1, IgG2b, IgG2c, and IgG3 Abs were assessed because mice with the C57BL/6 background lack the *Ighg2a* allele that codes for IgG2a but instead express IgG2c from the *Ighg2c* allele 33, 34. OVA‐specific IgE Abs were determined using a mouse IgE ELISA (OVA) kit (DS Pharma Biomedical Co., Ltd., Osaka, Japan). Concentrations of OVA‐specific IgE Abs in plasma were calculated using a standard curve.

### Body temperature monitoring

Mouse body temperature was measured between 9 and 11 a.m. Rectal temperature was measured with a digital thermometer (BAT‐12, Physitemp Instruments Inc., Clifton, NJ) equipped with a rectal probe (RET‐3, Physitemp Instruments Inc.) inserted to a depth of approximately 1.5 cm. The mice were lightly restrained during the insertion of the probe. To reduce methodological stress, mice were sufficiently acclimated to the procedure before the test period. All measurements were recorded when rectal temperature reading reached a plateau.

### β‐Hexosaminidase assay and determination of chemical mediators

MC/9 cells were used in degranulation assays. Cell culture conditions and methods of β‐hexosaminidase determination have been described in detail previously 12, 35. Concentrations of histamine, leukotriene B_4_ (LTB_4_), and prostaglandin D_2_ (PGD_2_) in each supernatant sample were determined using enzyme immunoassay kits (histamine: SPI‐Bio, Montigny le Bretonneux, France; LTB_4_ and PGE_2_: Cayman Chemical Co., Ann Arbor, MI, USA).

### Quantitative real‐time reverse transcription polymerase chain reaction

MC/9 cells were cultured with or without SF at various concentrations in Tyrode's salt solution. After 24 h, the cells were harvested, and total RNA was extracted from cells, using a Nucleo Spin RNA II Kit (Macherey‐Nagel GmbH & Co. KG, Düren, Germany). cDNA was synthesized from 500 ng of total RNA using SuperScript III (Life Technologies Co., Carlsbad, CA, USA) and oligo (dT) primers. One microliter of cDNA was subjected to real‐time reverse transcription polymerase chain reaction (RT‐PCR) using SYBR Green II (Takara Bio Inc., Shiga, Japan) with primers specific for tumor necrosis factor‐α (*Tnf*), interleukin‐4 (*Il4*), *Il5*, *Il10*, *Il13*, 36, chemokine (C‐C motif) ligand 5 (*Ccl5*) (Fwd‐5′ GCCAACCCAGAGAAGAAGTG and Rev‐5′ AGCAAGCAATGACAGGGAAG), and glyceraldehyde 3‐phosphate dehydrogenase (*Gapdh*) 37. Expression levels of mouse *Tnf*, *Ccl5*, *Il4*, *Il5*, *Il10*, *Il13*, and *Gapdh* genes were determined by real‐time RT‐PCR using an ABI 7500 system (Applied Biosystems Inc., Foster City, CA, USA).

### Statistical analysis

The titers of OVA‐specific Abs are expressed as the median with the interquartile range (IQR) 38. Comparison of values between two groups was made by the two‐tailed Mann–Whitney *U* test. Normally distributed variables were analyzed by the two‐tailed unpaired Student's *t*‐test for comparisons between groups, and the results are expressed as the mean ± standard deviation (SD). For comparison of more than two groups, one‐way analysis of variance (ANOVA) was performed. If the ANOVA reported a significant effect of the group, the Tukey's multiple comparisons test was performed as post hoc test. Two‐way repeated measures ANOVA (genotype: between‐subject factor; time: within‐subject factor) was performed to compare differences in body temperature of congenic normal and *W/W^v^* mice that were administered OVA with SF. If the ANOVA reported a significant effect of the group, the Bonferroni's multiple comparisons test was performed as post hoc test. Differences were considered significant if *P* < 0.05.

## Results

### Assessment of OVA‐specific humoral immunity in mast cell‐deficient mice

**Figure 1:**
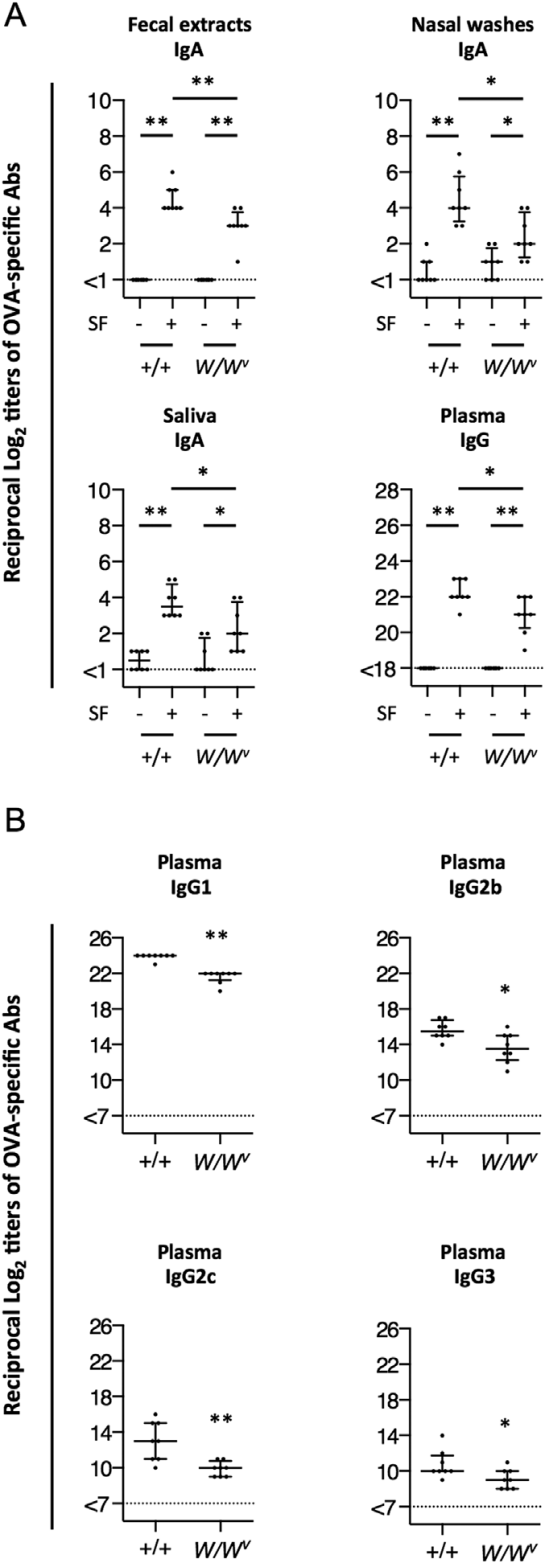
Ovalbumin‐specific antibody responses in mucosal secretions and plasma. Congenic normal (+/+) and *W/W^v^* mice were intranasally immunized with ovalbumin (OVA) with (+) or without (−) surfactin (SF) three times at weekly intervals. Mucosal secretions and plasma were collected at 1 week after the last immunization. OVA‐specific antibody (Ab) levels were determined by the endpoint enzyme‐linked immunosorbent assay. (A) Reciprocal log_2_ titers of OVA‐specific IgA Abs in the fecal extracts, nasal washes, and saliva, and IgG Abs in the plasma. (B) Reciprocal log_2_ titers of OVA‐specific IgG1, IgG2b, IgG2c, and IgG3 Abs in the plasma of normal (+/+) and *W/W^v^* mice immunized with OVA plus SF intranasally. Each dot represents a value from an individual mouse. Horizontal and vertical bars indicate the median with the interquartile range from two independent experiments (with eight mice in each experimental group). Significant differences between groups are indicated with asterisks as follows: *P < 0.05, **P < 0.005.

To evaluate the contribution of mast cells to SF adjuvanticity, the levels of OVA‐specific Abs in mucosal secretions (fecal extracts, nasal washes, and saliva) and plasma of *W/W^v^* and congenic normal mice were determined by endpoint ELISA. There were no significant differences in the titers of OVA‐specific IgA Abs in mucosal secretions and of IgG Abs in the plasma between *W/W^v^* and congenic normal mice intranasally immunized with OVA. These results indicated that the strength of OVA‐specific humoral immune responses was similar in *W/W^v^* and congenic normal mice immunized with OVA alone. In congenic normal mice, the titers of OVA‐specific Abs in both mucosal and systemic compartments were significantly enhanced by SF. Similar effects were observed in *W/W^v^* mice. Notably, the titers of OVA‐specific IgA Abs in mucosal secretions and IgG Abs in the plasma of *W/W^v^* mice were significantly lower than those in congenic normal mice (Fig. 1A). Likewise, significantly lower titers of OVA‐specific Abs in all IgG subclasses were observed in *W/W^v^* mice (Fig. 1B).

### Analysis of mast cell activation by SF

**Figure 2:**
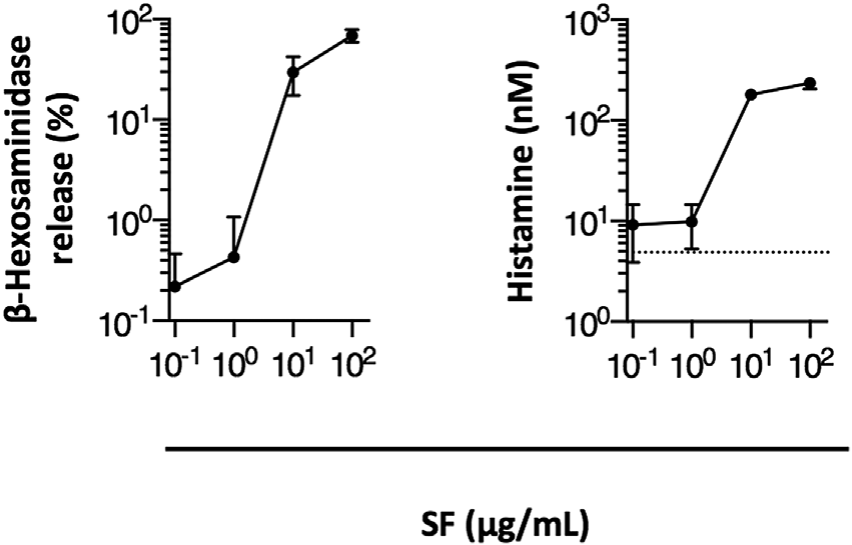
Degranulation of mast cells and histamine release evoked by surfactin. MC/9 cells were cultured with 10^−1^, 10^0^, 10^1^, or 10^2^ μg/mL SF for 30 min at 37°C. Left panel shows the relationship between surfactin (SF) concentration and the percentage of β‐hexosaminidase released in response to the stimulation with SF. Right panel shows the relationship between the concentration of histamine released into culture medium (determined in the supernatant) and SF concentration. Concentrations of histamine released from MC/9 cells were assessed by the enzyme immunoassay. Horizontal dashed lines denote the concentrations in the supernatants of MC/9 cells cultured without SF. Mean concentrations of histamine in culture supernatants without SF were 4.9 nM. Data represent the mean ± SD of three independent experiments.

Because significant differences in OVA‐specific Ab responses between *W/W^v^* and congenic normal mice were observed, we next analyzed the effects of SF on mast cells in vitro. The percentages of degranulated MC/9 cells were increased by SF in a concentration‐dependent manner, as measured by the β‐hexosaminidase assay. The mean fraction of β‐hexosaminidase released from cells treated with 100 μg/mL SF was 68.8% (Fig. 2A). Correspondingly, released histamine was also detected in the supernatants of MC/9 cells stimulated by SF (Fig. 2B).

### Measurements of body temperature

**Figure 3:**
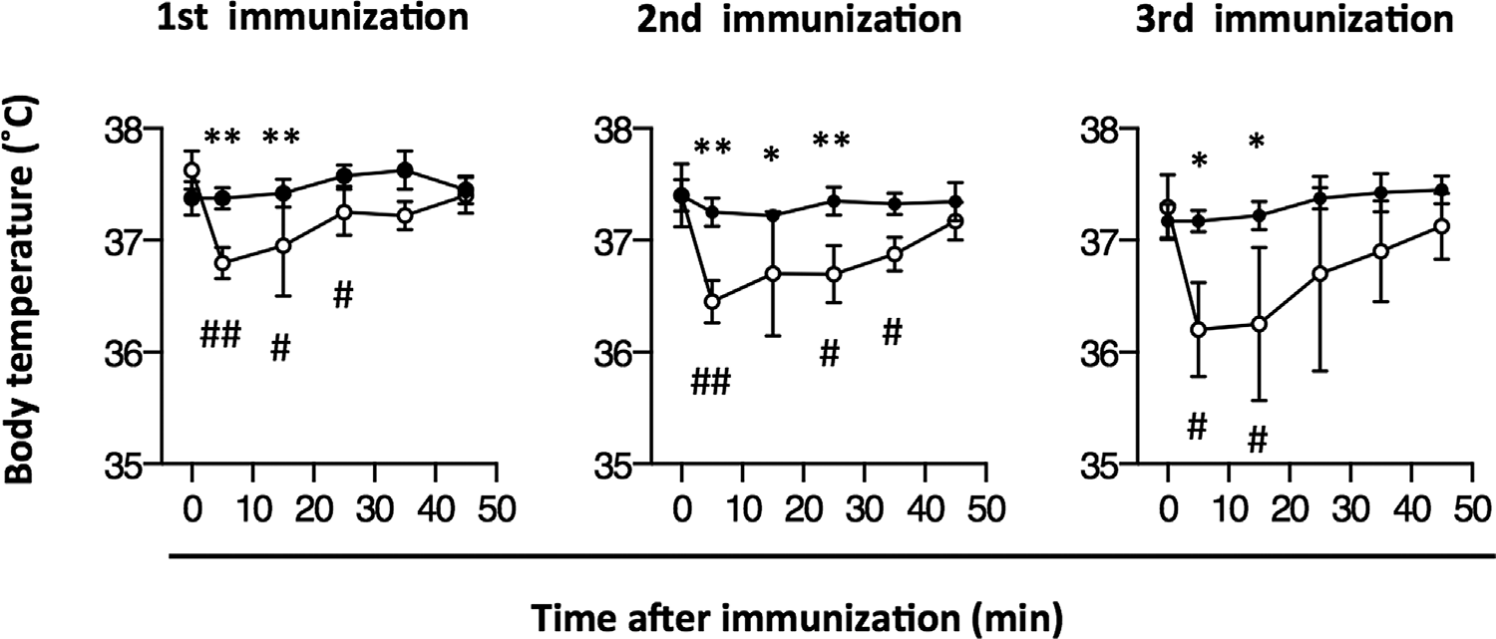
Changes in body temperature after immunization. Congenic normal (+/+) (open circles) and *W/W^v^* (closed circles) mice were intranasally immunized with ovalbumin (OVA) plus 500 μg of surfactin (SF) three times at weekly intervals. Rectal body temperatures were determined at 5, 15, 25, 35, and 45 min post immunization. Data represent the mean temperature ± SD of two independent experiments (with four mice in each experimental group). Significant differences between +/+ and *W/W^v^* mice are indicated with asterisks as follows: *P < 0.05, **P < 0.005. Significant differences between values measured at time 0 and other time points in congenic normal or *W/W^v^* mice are indicated with hash signs as follows: #*P* < 0.05, *##P* < 0.005.

Direct determination of degranulated mast cells in vivo is quite difficult 39. In general, the following pieces of evidence are thought to reflect selective mast cell activation in vivo: body temperature decrease, Evans blue extravasation, and/or ear swelling 40, 41. In mice intravenously injected with histamine, a decrease in body temperature was observed within 5 min, as has been reported previously 42. Hence, we measured body temperature to establish whether mast cells contributed to SF adjuvanticity. Temporal pattern of body temperature changes after intranasal immunization with OVA plus SF in congenic normal mice was significantly different from that in *W/W^v^* mice. These differences were observed at each of the three immunization rounds. Body temperature in congenic normal mice immunized with OVA plus SF dropped by approximately 1.0°C at 5 min after the immunization. This decrease in temperature was transient, with the lowest temperature being observed 5–15 min after immunization. The temperature gradually returned to almost normal temperature level in 35–45 min after immunization. In contrast, there were no significant decreases in body temperature in *W/W^v^* mice immunized with SF (Fig. 3).

### Determination of OVA‐specific IgE Abs

**Figure 4:**
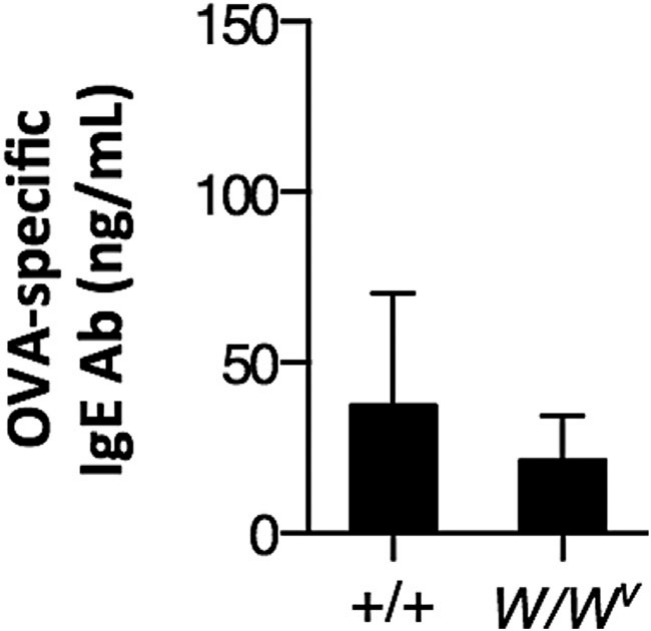
Ovalbumin‐specific IgE antibodies in the plasma. Congenic normal (+/+) and *W/W^v^* mice were intranasally immunized with ovalbumin (OVA) plus 500 μg of surfactin (SF) three times at weekly intervals. Plasma samples were collected at 1 week after the last immunization, and the concentrations of OVA‐specific IgE antibodies (Abs) were determined by the enzyme‐linked immunosorbent assay. Data represent the mean concentration ± SD of two independent experiments (with four mice in each experimental group).

Degranulation is mediated by binding of IgE Abs that interact with Ags to the Fcϵ receptor on the surface of mast cells 43. Accordingly, we assessed the levels of OVA‐specific IgE Abs in the plasma of immunized mice. OVA‐specific IgE Ab levels were below the detection limit (<2.7 ng/mL) in the plasma of *W/W^v^* and congenic normal mice before immunization (data not shown). The levels of OVA‐specific IgE Abs in the plasma of congenic normal and *W/W^v^* mice were 32.7 ± 33.0 and 21.4 ± 13.1 ng/mL, respectively (Fig. 4). No significant differences in OVA‐specific IgE Ab levels were observed between the groups at 1 week after the last immunization. From the experiments described above, we concluded that SF directly activates mast cell in vivo.

### Induction of chemical mediator release and cytokine mRNA expression by SF

**Figure 5:**
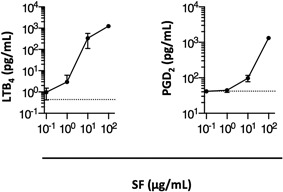
Concentrations of chemical mediators in culture supernatants. MC/9 cells were cultured with 10^−1^, 10^0^, 10^1^, or 10^2^ μg/mL surfactin (SF) for 30 min at 37°C. Concentrations of LTB_4_ (left panel) or PGD_2_ (right panel) released from MC/9 cells were assessed by the enzyme immunoassay. Horizontal dashed lines denote the concentrations in the supernatants of MC/9 cells cultured without SF. Mean concentrations of LTB_4_ and PGD_2_ in culture supernatants without SF were 0.44 pg/mL and 41.8 pg/mL, respectively.

Next, we analyzed the relationship between mast cell activation and enhancement of humoral immune responses induced by SF. Mast cells contribute to the induction of acquired immune responses by producing various chemical mediators and cytokines 3, 44, 45. Therefore, we assessed the released amounts of typical chemical mediators and expression of typical cytokines, associated with acquired immunity, in mast cells 3, 46. We revealed that the levels of the chemical mediators LTB_4_ and PGD_2_ were significantly elevated in the supernatants of MC/9 cells stimulated by SF (Fig. 5).

**Figure 6:**
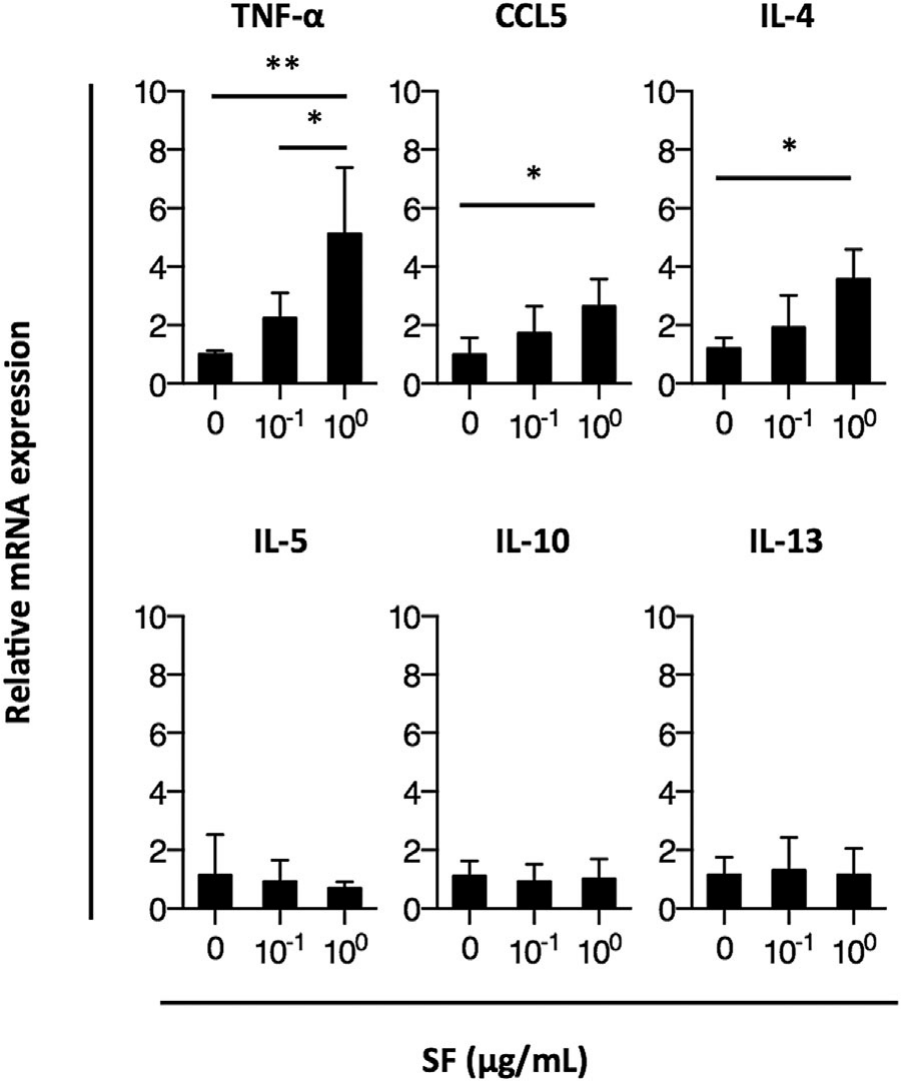
Expression levels of cytokine mRNAs in MC/9 cells. The cells were cultured with 0, 10^−1^, or 10^0^ μg/mL surfactin (SF) for 24 h at 37°C. Quantitative real‐time RT‐PCR was performed to compare *Tnf*, *Ccr5*, *Il4*, *Il5*, *Il10*, and *Il13* mRNA expression levels in MC/9 cells. Results are presented as fold change compared to respective levels in cells that did not undergo SF stimulation. Data represent the mean ± SD of three independent experiments. Significant differences are indicated by asterisks as follows: *P < 0.05, **P < 0.005.

Stimulation by SF significantly enhanced mRNA expression levels of *Tnf*, *Ccl5*, and *Il4* genes. In particular, the expression level of *Tnf* mRNA was elevated 5.1‐fold following a treatment with 1 μg/mL SF as compared to *Tnf* mRNA level in negative control cells. Expression levels of *Il5*, *Il10*, and *Il13* mRNAs were not changed by SF stimulation (Fig. 6). Thus, augmented levels of the chemical mediators LTB_4_ and PGD_2_, as well as increased levels of *Tnf*, *Ccl5*, and *Il4* mRNA likely contributed to the adjuvanticity of intranasally administered SF in immunized mice.

## Discussion

In the present study, we investigated properties of SF as a mucosal adjuvant, using mast cell‐deficient mice, and demonstrated that SF adjuvanticity is partially facilitated by mast cell activation. For many adjuvants, mast cell activation is not an obligatory condition of their adjuvanticity. However, adjuvanticity of mast cell activators, such as compound 48/80 or IL‐18, was shown to be significantly reduced in *W/W^v^* mice compared to that in congenic normal mice 6, 47.

The mechanisms of mast cell activation are divided broadly into two categories: immunologic and non‐immunologic mechanisms 48. Immunologic mast cell activation, exemplified by systemic anaphylaxis, is an IgE Ab‐dependent process 48, 49, 50, 51, 52, 53. However, mast cell activators, including compound 48/80 and PMB, directly activate mast cells 10, 11, 54. In our experiments, SF induced degranulation and release of histamine from mast cells, as shown in Figure 2. These observations revealed that the activation of mast cells by SF was an IgE Ab‐independent process in vitro.

To investigate whether mast cells were activated by SF in vivo, we measured body temperature of mice immunized with OVA plus SF intranasally. This treatment decreased body temperature in congenic normal mice. Notably, we observed the decrease in body temperature in congenic normal mice already after the first immunization. OVA‐specific IgE Abs were not detected in the plasma of naïve congenic normal mice. The concentration of OVA‐specific IgE Abs in the plasma of congenic normal mice immunized with OVA plus SF was 37.4 ng/mL. In some studies that examined allergy caused by OVA sensitization in mouse models, the values of OVA‐specific IgE Abs were 300–3,000 ng/mL 49, 50, 51, 52, 53. IgE Ab concentrations in mice immunized with OVA plus SF in our experiments were apparently lower than those observed in OVA sensitization experiments. Additionally, although body temperature time‐courses were significantly different in *W/W^v^* and congenic normal mice, there were no significant differences in the levels of OVA‐specific IgE Abs between genotypes. This finding indicates that body temperature decrease was not directly associated with the binding of OVA‐specific IgE Abs to mast cells in congenic normal mice. Furthermore, a decrease in body temperature has been reported in BALB/c mice administered with compound 48/80 55, which is in agreement with our results. Consequently, although we could not directly demonstrate degranulation of mast cells by SF in mouse nasal mucosa, SF probably stimulated mast cells whose activation likely contributed to SF adjuvanticity in vivo.

One of the important criteria for the development of mucosal adjuvants should be their high potential to induce Ag‐specific mucosal IgA and plasma IgG Ab responses, as well as low propensity to trigger IgE Ab production. Indeed, some children vaccinated with certain live virus vaccines are prone to anaphylaxis 56. Furthermore, it has been reported that cholera toxin, as a mucosal adjuvant, increased the risk of IgE‐mediated inflammation and allergy 57, 58. Our results suggest that mast cell activation by SF did not induce IgE Ab production. Therefore, IgE Ab‐independent action of SF is advantageous for the development of SF as an adjuvant.

As compared with the data from previous studies of adjuvanticity of SF and PMB 12, 16, the titers of OVA‐specific IgG Abs in the plasma of mice immunized with SF were significantly higher than those of mice immunized with PMB (*P* < 0.0005), when C57BL/6 mice were immunized with 5 μg of SF or PMB plus OVA by the same immunization protocol. In the assessments of MC/9 cell degranulation, the mean percentages of β‐hexosaminidase released by applications of 10 μg/mL of SF or PMB were 29.8% and 4.8%, respectively. SF released significantly more β‐hexosaminidase than did PMB (*P* < 0.05). We reasoned that the extent of mast cell activation and adjuvanticity of compounds such as SF and PMB might be interrelated. One major difference between SF and PMB chemical structures is the length of alkyl chain: 11 and 6–7 carbon atoms, respectively 21. Indeed, when the release of histamine from mast cells was induced by parabens with different alkyl chains, ranging from methylparaben to dodecylparaben, a complex relationship was observed between the extent of mast cell activation and the length of alkyl chain 59. Structural differences between SF and PMB likely explain higher adjuvanticity of SF mediated by mast cell activation.

Next, we examined whether mast cell activation by SF mediated the enhancement of acquired immune responses. Our experiments showed that histamine, LTB_4_, and PGD_2_ were released from mast cells by SF. Mast cells participate in adaptive immunity through the expression and secretion of several pro‐inflammatory factors 5, 6. Histamine promotes Ag uptake and cross‐presentation 60 and enhances the up‐regulation of co‐stimulatory molecules required for T‐cell activation on dendritic cells 61. LTB_4_ induces NLRP3 inflammasome activation 62. PGD_2_ induces recruitment of T helper type 2 (Th2) cells and Th2 cytokine synthesis 63, 64.

OVA‐specific IgG1 Abs in the plasma of immunized mice were dominant among the IgG subclasses. The relationship between IgG1 and IgG2 levels is a surrogate marker for Th1 and Th2 cell population balance 65. We observed that the mean ratio of OVA‐specific IgG1 and IgG2c Ab in congenic normal mice was 1.2‐fold higher than that in *W/W^v^* mice (Fig. 1b). Although IgG1/IgG2c ratios between *W/W^v^* and congenic normal mice were not statistically different, this indicated that Th2 immune responses in congenic normal mice were more predominant than that in *W/W^v^* mice. It is conceivable that mast cell‐derived factors induced by SF might evoke OVA‐specific, primarily Th2 type immune responses.

Furthermore, we found that SF up‐regulated mRNA expression levels of some cytokines. Different aspects of acquired immune response are mediated by the cytokines released from mast cells. TNF‐α promotes dendritic cell activation, Ag presentation, and the influx of dendritic cells into draining lymph nodes 61, 66. Mast cell‐derived IL‐4 and CCL5 promote macrophage activation 67 and T cell recruitment 68, respectively. Release of these cytokines likely contributed to SF adjuvanticity.

We considered whether the mechanism of SF action was similar to that of other adjuvants. Alum and lipopolysaccharide (LPS) have potent adjuvanticity 69, 70 and can activate mast cells 71, 72. Mast cells stimulated by alum release histamine and produce IL‐5. However, using mast cell‐deficient mice, it was demonstrated that mast cells were not required for the augmentation of adaptive immune responses by alum 72. In MC/9 cells, LPS induced the expression of IL‐5, IL‐10, and IL‐13, but not that of IL‐4 71. As can be seen from our data, cytokine profiles following exposure to SF were different from those seen after treatments with other adjuvants. Therefore, we suggest that the properties of SF adjuvanticity are distinct from those of adjuvanticity of alum and LPS.

Our previous study showed that SF strongly reinforced humoral immune responses at nasal mucosa. The numbers of OVA‐specific IgA and IgG Ab‐forming cells (AFCs) in the nasal lamina propria (n‐LP), nasopharynx‐associated lymphoid tissues (NALTs), small intestinal lamina propria (i‐LP), and spleens of mice intranasally immunized with OVA plus SF were significantly higher than those in mice immunized with OVA alone. In particular, the numbers of OVA‐specific IgA AFCs in nasal mucosal tissues (n‐LP and NALTs) were significantly higher in mice immunized with OVA plus SF 16. The maximum potentiation of immune responses by SF was observed in the nasal mucosa. In the current study, medians of OVA‐specific IgA Ab titers in nasal washes varied greatly between genotypes, although the role of mast cells to induce Ag‐specific humoral immune responses could be of marginal significance, as shown in Figure 1.

These previous and current observations indicated that mast cell activation should be one of the mechanisms affecting SF adjuvanticity. However, OVA‐specific Ab did not entirely disappear in *W/W^v^* mice immunized with OVA plus SF. It is well‐known that the activation of inflammasomes can affect adaptive immune responses 73. WH1fungin, one of SF family members, activated mitogen‐activated protein kinases, nuclear factor‐κB, and inflammasomes in macrophages in vitro 74. It was also reported that PMB activated NLRP3 inflammasomes in dendritic cells in vitro 75. Those studies suggested that SF might activate inflammasomes, and it is conceivable that the activation of inflammasomes may be another mechanism contributing to the adjuvanticity of SF. Moreover, the proportion of γδ T cells in the splenocytes of *W/W^v^* mice has been shown to be lower than that in congenic normal mice 76. γδ T cell‐deficient mice had impaired mucosal IgA Ab responses 77. Thus, the contribution of γδ T cells to SF adjuvanticity cannot be ruled out.

In conclusion, by both in vivo and in vitro experiments, we showed that mast cell activation is concomitant with the manifestation of SF adjuvanticity. Although some mechanisms of SF adjuvanticity remain unknown, our data suggest that activation of mast cells likely partially contributes to mucosal adjuvanticity of SF. As mast cells are abundant in the nasal mucosa 78, our results explain the efficacy of intranasal administration of SF as adjuvant.

## Acknowledgments

We thank Sumiko Yaegashi (School of Medicine, Iwate Medical University) for technical assistance, as well as Wataru Yasuno, Miyuki Takahashi, and Tomoki Takahashi (Center for In Vivo Sciences, Iwate Medical University) for animal care. We would like to thank Editage (www.editage.jp) for English language editing. The funders had no role in study design, data collection and analysis, decision to publish, or preparation of the manuscript.

## Conflict of Interest

The authors declare that they have no conflict of interest.
